# Pathogenicity of *Citrobacter freundii* Causing Mass Mortalities of *Macrobrachium rosenbergii* and Its Induced Host Immune Response

**DOI:** 10.3390/microorganisms12102079

**Published:** 2024-10-17

**Authors:** Anting Chen, Qieqi Qian, Xiaoyu Cai, Jia Yin, Yan Liu, Qi Dong, Xiaojian Gao, Qun Jiang, Xiaojun Zhang

**Affiliations:** College of Animal Science and Technology, Yangzhou University, Yangzhou 225009, China; chenanting2002@163.com (A.C.); qieqi2000810@163.com (Q.Q.); 18136877121@163.com (X.C.); 15189615905@163.com (J.Y.); 15062801209@163.com (Y.L.); 15952707958@163.com (Q.D.); gaoxj336@163.com (X.G.); jiangqun1013@163.com (Q.J.)

**Keywords:** *Macrobrachium rosenbergii*, *Citrobacter freundii*, virulence, immune-related genes

## Abstract

*Citrobacter freundii* is an opportunistic pathogen of freshwater aquatic animals, which severely restricts the sustainable development of the aquaculture industry. In this study, a dominant strain, named FSNM-1, was isolated from the hepatopancreas of diseased *Macrobrachium rosenbergii*. This strain was identified as *C. freundii* based on a comprehensive analysis of its morphological, physiological, and biochemical features and molecular identification. Challenge experiments were conducted to assess the pathogenicity of *C. freundii* to *M. rosenbergii*. The results showed that the FSNM-1 strain had high virulence to *M. rosenbergii* with a median lethal dose (LD_50_) of 1.1 × 10^6^ CFU/mL. Histopathological analysis revealed that *C. freundii* infection caused different degrees of inflammation in the hepatopancreas, gills, and intestines of *M. rosenbergii*. The detection of virulence-related genes revealed that the FSNM-1 strain carried colonization factor antigen (*cfa1*, *cfa2*), ureases (*ureG*, *ureF*, *ureD*, *ureE*), and outer membrane protein (*ompX*), and virulence factor detection showed that the FSNM-1 strain had lecithinase, amylase, lipase, gelatinase, and hemolysin activities but did not produce protease and DNase activities. To investigate the immune response of *M. rosenbergii* to *C. freundii*, the expression levels of *ALF3*, *MyD88*, *SOD*, *proPO*, *TRAF6*, and *TNF* immune-related genes were monitored at different points of time in the hepatopancreas, gills, intestines, and hemocytes of *M. rosenbergii* after infection. The results demonstrated a significant upregulation in the expression levels of the *ALF3*, *MyD88*, *SOD*, *proPO*, *TRAF6*, and *TNF* genes in *M. rosenbergii* at the early stage of *C. freundii* infection. This study highlights *C. freundii* as a major pathogen causing mass mortality in *M. rosenbergii* and provides valuable insights into its virulence mechanisms and the host’s immune response.

## 1. Introduction

The giant freshwater prawn *Macrobrachium rosenbergii* is one of the most economically important crustaceans widely farmed in China and other Southeast Asian countries [1]. According to FAO reports, global production of *M. rosenbergii* reached 327,828 tons in 2023 [2]. Since being introduced in Japan in 1976, *M. rosenbergii* has evolved into a specialized aquaculture species with substantial production output and promising development prospects in China [3], where its widespread cultivation is attributed to its nutritional value, superior meat quality, and rapid growth [4]. Nevertheless, the escalating stocking densities and deteriorating environmental conditions have led to frequent disease outbreaks, resulting in high mortality rates and significant economic losses in prawn aquaculture [5]. Disease outbreaks caused by various pathogens, including bacteria, viruses, rickettsia, fungi, protozoa, and other eukaryotic microorganisms, represent a major threat to the global prawn farming industry [6]. Various bacterial diseases negatively impacting the health of *M. rosenbergii* had been reported, including *Enterobacter cloacae* [7], non-O1/O139 *Vibrio cholerae* [8], *Vibrio vulnificus* [9], *Vibrio parahaemolyticus* [10], *Aeromonas hydrophila* [11], *Aeromonas dhakensis* [12], and *Aeromonas veronii* [13]. Furthermore, the incidence associated with viral diseases has been increasing, such as *M. rosenbergii* nodavirus (*Mr*NV) [14], extra-small virus (XSV) [15], infectious precocity virus (IPV) [16], covert mortality nodavirus (CMNV) [17], white spot syndrome virus (WSSV) [18], infectious hypodermal and hematopoietic necrosis virus (IHHNV) [19], and Decapod iridescent virus 1 (DIV1) [20]. Since June 2022, mass mortality outbreaks of *M. rosenbergii* have taken place on farms in Gaoyou County, Yangzhou City, Jiangsu Province, leading to substantial economic losses in *M. rosenbergii* aquaculture. In our study, *Citrobacter freundii* was isolated from diseased prawns and identified as the main pathogen.

*Citrobacter freundii* is a facultative anaerobic, Gram-negative, non-spore-forming, rod-shaped bacterium of the Enterobacteriaceae family and the *Citrobacter* genus [21]. In recent years, this opportunistic pathogen has been implicated in diseases affecting many types of aquatic products, such as fish, shrimp, and crabs [22]. Infection with *C. freundii* caused a range of symptoms across various species; for instance, *Eriocheir sinensis* exhibited septicemia, black gill disease, and hepatopancreatic necrosis [23], *Acipenser sinensis* showed ventral surface hyperemia [24], *Oreochromis mossambicus* displayed septicemia and hemorrhage [25], *Carassius auratus* experienced cutaneous hemorrhages [26], and *Micropterus salmoides* suffered from liver hemorrhage and spleen enlargement [27]. Moreover, *C. freundii* also can infect *Labeo rohita* [28], *Rhamdia quelen* [29], *Potamotrygon motoro* [30], *Procambarus clarkii* [31], and *Macrobrachium nipponense* [32]. *C. freundii* infections have caused substantial commercial losses in the global aquaculture industry. However, reports of *C. freundii* infections leading to disease outbreaks in *M. rosenbergii* are limited.

In this study, the pathogenicity of *C. freundii* to *M. rosenbergii* was investigated through artificial challenge tests, histopathological analysis, and the detection of virulence-related genes and virulence factors of the strain. Additionally, the immune response mechanisms of *M. rosenbergii* infected with *C. freundii* were investigated by determining the relative expression levels of immune-related genes. These findings will enhance the understanding of the susceptibility and immune response in *M. rosenbergii* after *C. freundii* infection.

## 2. Materials and Methods

### 2.1. Bacterial Isolation

In June 2022, mass mortality outbreaks occurred among cultured *M. rosenbergii* on a farm in Gaoyou County, Jiangsu Province, China. The affected prawns exhibited clinical symptoms of soft-shell, lethargic swimming, loss of appetite, and whitish muscle. Diseased prawns were sterilized with 75% ethanol and dissected using sterile instruments. Hepatopancreas tissues were sampled and inoculated onto LB agar plates, followed by incubation at 28 °C for 24 h, and the dominant uniform isolates were purified. The representative strain, designated FSNM-1, was preserved in an LB liquid medium supplemented with 30% (*v*/*v*) sterile glycerol and stored at −80 °C.

### 2.2. Bacterial Virulence Assay

The experimental *M. rosenbergii* (3.12 ± 0.13 g) was acquired from a farm in Gaoyou County, Jiangsu Province, China. Before the challenge experiment, the prawns were acclimated for one week in aerated, filtered water at 28 °C. To ensure their health, the prawns underwent random bacterial isolation and were verified to be free of virus infections by PCR assay using specific primers [20]. Then *M. rosenbergii* were divided into six groups, with each group replicated in triplicate. Each group was kept in a 90 L tank containing 20 prawns, with the water temperature maintained at 26 ± 2 °C and dissolved oxygen levels at 5.0 mg/L. The FSNM-1 strain was inoculated into LB liquid medium at 28 °C with shaking at 180 rpm for 18 h. The bacterial solution was diluted into five concentrations using a gradient with sterile saline: 1.8 × 10^8^, 1.8 × 10^7^, 1.8 × 10^6^, 1.8 × 10^5^, and 1.8 × 10^4^ CFU/mL. The infected groups were administered the bacterial solutions at the specified concentrations (50 μL per prawn) by intramuscular injection, while the control group received sterile saline. Over a period of 14 days, mortalities and clinical symptoms of the infected prawns were systematically observed and recorded. Additionally, deceased prawns were subjected to bacterial isolation and identification. The median lethal dose (LD_50_) was calculated by assessing cumulative experimental mortality through the Behrens and Karber method [33].

### 2.3. Histopathology

Tissues from the hepatopancreas, gills, and intestines of both infected and control groups were fixed in Bouin’s solution for histopathological analysis. The histopathological examination using the Behera et al. method [28]. Fixed tissue samples underwent dehydration in an ethanol series, clearing in xylene, and embedding in paraffin wax. The paraffin blocks were trimmed and sectioned using standard techniques, after which the sections were stained with Hematoxylin and Eosin (H&E) and examined under light microscopy.

### 2.4. Identification of Bacteria

Transmission electron microscopy (TEM) was utilized to examine the FSNM-1 strain. Initially, cells were collected by centrifugation (5000 rpm, 15 min, 4 °C), then washed twice with sterilized PBS (pH 7.4). The cells were subsequently fixed in 2.5% glutaraldehyde, post-fixed with osmium tetroxide, dehydrated through a graded ethanol series, and coated with a gold-palladium alloy. Observations were made using a Zeiss EM10 transmission electron microscope (Zeiss, Oberkochen, Germany), allowing for analysis of flagella types and sizes. In addition, physiological and biochemical assays were conducted on the bacterial culture using standard plate and tube tests (Tianhe Microorganism Reagent Co., Ltd., Hangzhou, China), as outlined in Bergey’s Manual of Systematic Bacteriology [34]. Total genomic DNA from the FSNM-1 strain was extracted using the EasyPure Genomic DNA Kit (TransGen Biotech, Beijing, China) following the provided protocol. The *gyrB* gene sequence of the FSNM-1 strain was amplified by PCR as described by Zhang et al. [35]. The PCR products were subsequently sequenced at Nanjing Tsingke Biotechnology Co., Ltd., Nanjing, China. Sequence alignments were performed using the MEGA7.0, and a phylogenetic tree was constructed using the neighbor-joining (N-J) method. The reliability of the tree topology was assessed with 1000 bootstrap replications.

### 2.5. Determination of Extracellular Enzymes and Hemolysin

The determination of extracellular enzymes and hemolysin using the Peng et al. method [12]. Hemolysin, caseinase, amylase, gelatinase, lipase, and lecithinase activities of the FSNM-1 strain were evaluated on nutrient agar supplemented with 7% rabbit erythrocytes, 2.5% skimmed milk, 2% starch, 1% gelatin, 1% Tween-80, and 10% egg yolk, respectively. Briefly, 5 μL of FSNM-1DNA were spot-inoculated onto these agar plates and incubated at 28 °C for 24 h. The presence of lytic halo around the colonies indicated the activity of the respective extracellular enzymes, and the diameters of these halos were measured. All tests were conducted in triplicate.

### 2.6. Determination of Virulence-Related Genes

The FSNM-1 strain was screened for the presence of colonization factor antigens (*cfa1*, *cfa2*), outer membrane protein (*ompX*), and urease genes (*ureD*, *ureE*, *ureF*, *ureG*) to evaluate its pathogenic potential. Genomic DNA was extracted from the isolated strain using the EasyPure Genomic DNA Kit (TransGen Biotech, Beijing, China), with sterile PCR-grade water serving as a negative control. Specific primers used in the PCR analysis are listed in Table 1.

### 2.7. Detection of Immune-Related Genes Expression After C. freundii Infection

The expression pattern of immune genes in the hepatopancreas, gills, intestines, and hemocytes of *M. rosenbergii* was monitored at different points of time after *C. freundii* infection. The determination of immune-related gene expression after *C. freundii* infection using the Gao et al. method [8]. For the immune challenge assays, 100 prawns (mean body weight 3.12 ± 0.13 g) were exposed to *C. freundii* at a final concentration of 10^6^ CFU/mL. The control group was cultured in filtered fresh water devoid of *C. freundii*. Tissue samples from the hepatopancreas, gills, intestines, and hemocytes were collected at 0, 6, 12, 24, 48, 72, and 96 h post-infection (hpi). For each time point, hepatopancreas, gills, intestines, and hemocytes were collected, and three individuals were collected to generate a mixed sample.

Total RNA from hepatopancreas, gills, intestines, and hemocytes was extracted with TRIzol reagent (TIANGEN, Beijing, China). For first-strand cDNA synthesis, 1 μg of RNA was reverse transcribed using FastKing-RT SuperMix (TIANGEN, Beijing, China). Immune-related genes, including Anti-lipopolysaccharide factor-3 (ALF3), tumor necrosis factor receptor-associated factor 6 (TRAF6), tumor necrosis factor (TNF), prophenoloxidase (Propo), superoxide dismutase (SOD), and myeloid differentiation primary response 88 (MyD88), were quantified by qRT-PCR. The qRT-PCR was conducted with FastFire qPCR PreMix SYBR Green (TIANGEN, Beijing, China) on a Roche LightCycler^®^ Real-Time PCR System. The primer sequences used are shown in Table 2. Three replicates of each gene were performed, with primer specificity confirmed by melting curve analysis. 18S rRNA served as the reference gene, and relative expression levels were calculated using the 2^−ΔΔCt^ method. Statistical significance was assessed by one-way ANOVA with SPSS version 17.0. These results are presented as means ± standard deviation (SD), with differences considered significant at *p* < 0.05 and extremely significant at *p* < 0.01.

## 3. Results

### 3.1. Virulence of the Isolate

**Figure 1:**
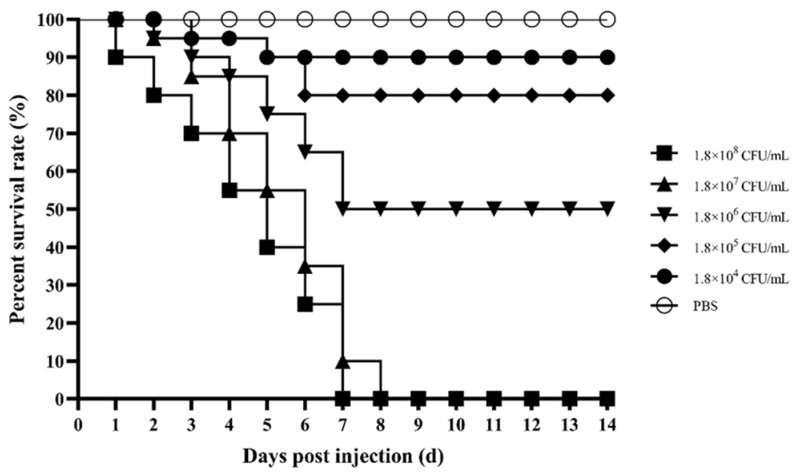
Pathogenicity of the FSNM-1 strain to *M. rosenbergii*.

The pathogenicity results of the isolated strain of *M. rosenbergii* are shown in Figure 1. Complete mortality of *M. rosenbergii* occurred within 7 days after infection with 1.8 × 10^8^ CFU/mL. The mortality rates of *M. rosenbergii* exposed to the FSNM-1 strain at concentrations of 1.8 × 10^7^, 1.8 × 10^6^, 1.8 × 10^5^, and 1.8 × 10^4^ CFU/mL were 90%, 50%, 20%, and 10%, respectively, with no fatalities observed in the control group within 14 days. The calculated LD_50_ to *M. rosenbergii* was 1.1 × 10^6^ CFU/mL. Additionally, bacteria re-isolated from the infected *M. rosenbergii* were both phenotypically and molecularly confirmed as the FSNM-1 strain.

### 3.2. Histopathological Changes

**Figure 2:**
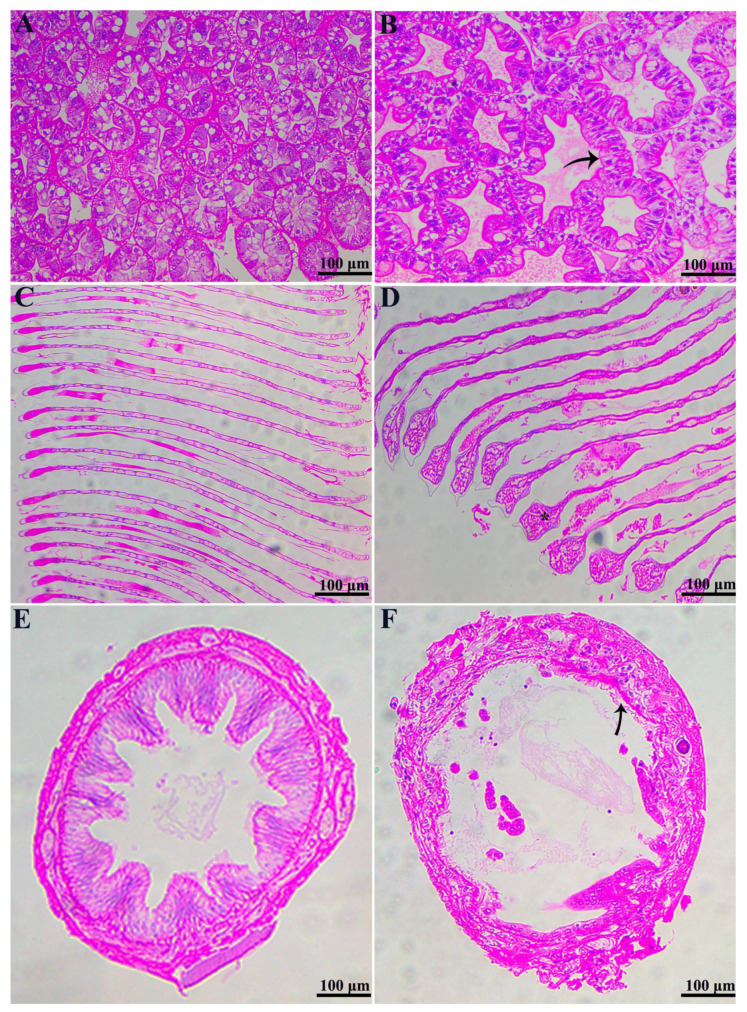
H&E-stained histological sections of *M. rosenbergii* (bar = 100 μm). (**A**) Hepatopancreas of the control group; (**B**) Hepatopancreas of the test group, the arrow shows loss of the star-like shape of the lumen; (**C**) gills of the control group; (**D**) gills of the test group, * shows clubbing at the tip of the gill filaments; (**E**) Intestine of the control group; (**F**) Intestine of the test group.

The infection of *C. freundii* resulted in significant histological alterations in the hepatopancreas, gills, and intestines of *M. rosenbergii*, with no lesions observed in normal tissues (Figure 2A,C,E). In the infected group, hepatic tubules exhibited disorganization, significant gaps, and pronounced inflammation. The luminal structure was severely deformed, the walls were markedly thinned, vacuolation was extensive, and localized hepatocytes and connective tissue were fractured and eroded, leading to irreversible damage (Figure 2B). Furthermore, the gills of the infected group exhibited a considerable accumulation of inflammatory cells within the gill arches, accompanied by hyperemia, edema, and disorganization of the gill filaments (Figure 2D). Moreover, the intestinal epithelium in the infected group exhibited severe detachment from the basement membrane, with the intestinal villi showing irregular arrangement, disruption, or dissolution (Figure 2F).

### 3.3. Characterization and Identification of Isolate FSNM-1

**Figure 3:**
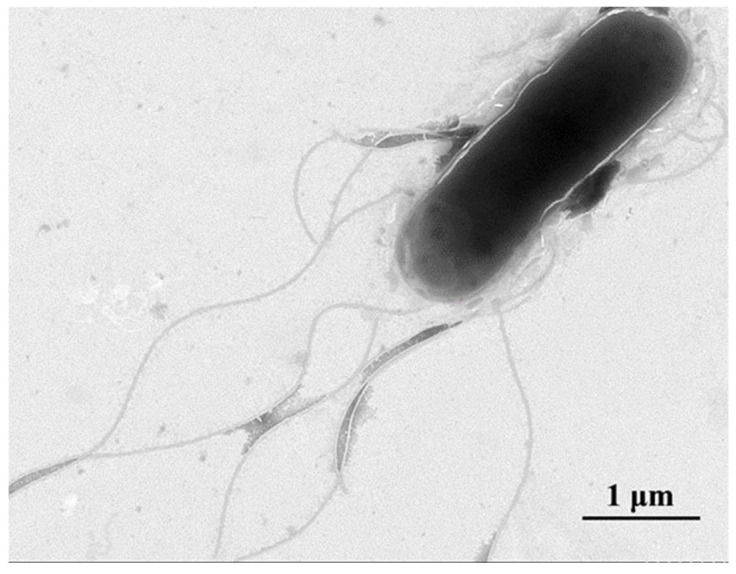
Electron micrograph of the FSNM-1 strain showing peri-flagellum (bar = 1 μm).

TEM analysis showed that the FSNM-1 strain was a rod-shaped bacterium with rounded ends and dimensions of approximately 0.8–1.0 μm in width and 2.0–2.5 μm in length. It lacked bacterial hairs or pods and exhibited motility via peritrichous flagella. (Figure 3).

In addition, the FSNM-1 strain was positive for motility, mannose, sorbose, mannitol, arabinose, nitrate reduction, citrate utilization, β-galactosidase, tartrate utilization, inulin, xylose, and maltose. Additionally, the FSNM-1 strain was negative for phenylalanine, malonate utilization, DNase, inositol, arabinol, adonitol, and gelatin hydrolysis. (Table 3). The FSNM-1 strain exhibited characteristics matching those of *C. freundii* as described in Bergey’s Manual of Systematic Bacteriology [34].

**Figure 4:**
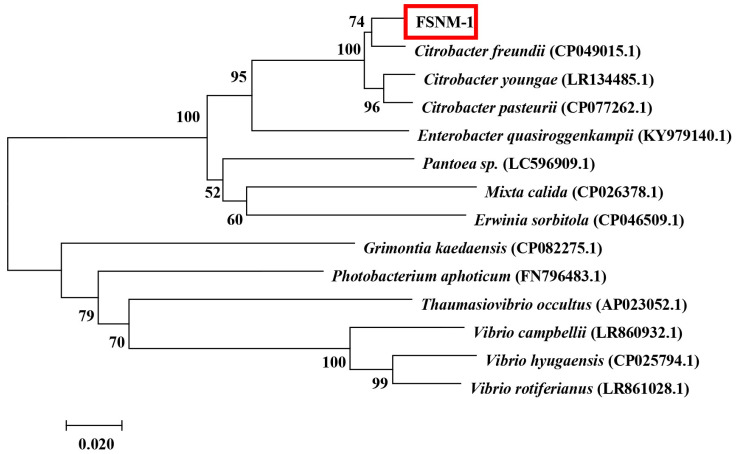
Neighbor-joining (FSNM-1) phylogenetic tree based on partial *gyrB* gene sequences. Bootstrap values are shown beside the clades.

The *gyrB* gene sequences of the FSNM-1 strain were determined and deposited in GenBank under accession number OQ722444. BLAST analysis revealed a 99% similarity with the *gyrB* gene of *C. freundii* (accession number: KM509081, MW199734). A phylogenetic tree based on *gyrB* gene sequences showed the FSNM-1 strain clustering with *C. freundii* strains (Figure 4) with a high bootstrap value, strongly supporting the classification of the FSNM-1 strain as *C. freundii*.

### 3.4. Virulence Genes of Isolate FSNM-1

**Figure 5:**
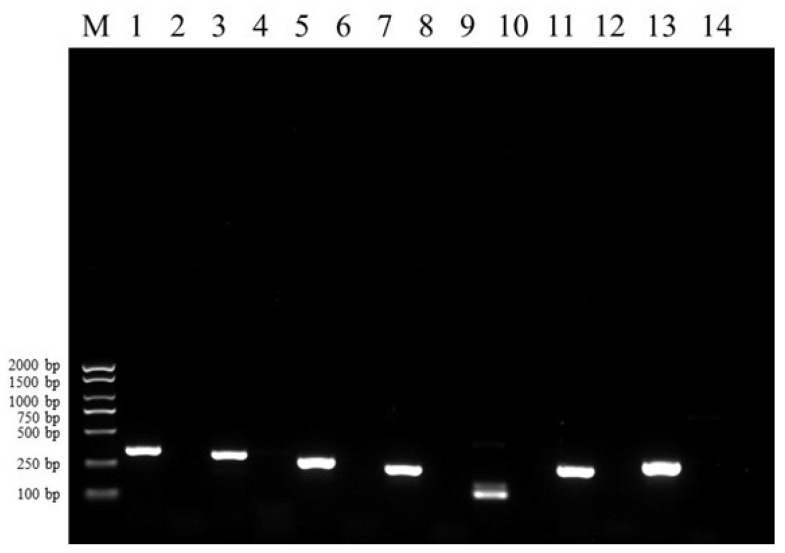
Agarose (1%) gel electrophoresis of PCR virulence gene products. M: 2000 bp DNA marker, Lane 1: *cfa1*, 2: negative control, 3: *cfa2*, 4: negative control, 5: *ompX*, 6: negative control, 7: *ureD*, 8: negative control, 9: *ureG*, 10: negative control, 11: *ureE*, 12: negative control, 13: *ureF*, 14: negative control.

Figure 5 illustrates that the FSNM-1 strain carried colonization factor antigen (*cfa1*, *cfa2*), ureases (*ureG*, *ureF*, *ureD*, *ureE*), and outer membrane protein (*ompX*) virulence-related genes. Additionally, no fragments were identified in the negative control.

### 3.5. Detection of Extracellular Enzymes and Hemolysin Activities

The hemolysin activity and extracellular enzyme detection revealed that the FSNM-1 strain produced lecithin, lipase, amylase, gelatinase, and hemolysin (Table 4) but did not exhibit protease or DNase activities.

### 3.6. Expression Analysis of Immune-Related Genes

#### 3.6.1. Immune-Related Genes Expression in Hepatopancreas After *C. freundii* Infection

**Figure 6:**
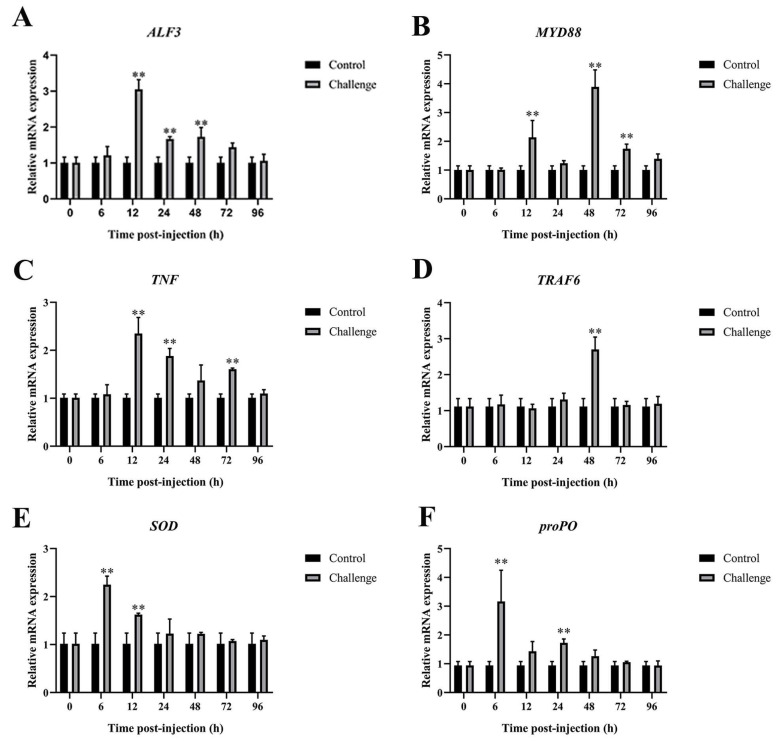
Immune-related gene expression in hepatopancreas after *C. freundii* infection. (**A**) *ALF3*, (**B**) *MyD88*, (**C**) *TNF*, (**D**) *TRAF6*, (**E**) *SOD*, (**F**) *proPO*. Data presented as mean ± SD, ** *p* < 0.01.

The expression of immune-related genes in the hepatopancreas is illustrated in Figure 6. *ALF3* and *TNF* were markedly upregulated at 12 hpi, with maximum levels of 3.02-fold and 2.33-fold, respectively. The gene expression levels of *TRAF6* rapidly increased after infection and reached a maximum of 48 hpi, with 2.41-fold increases compared to the control group. At 12 hpi, *MyD88* was upregulated, reaching its highest expression level at 48 hpi with a 3.85-fold increase compared to the control. *SOD* and *proPO* were significantly upregulated at 6 hpi, with peak increases of 2.23-fold and 3.37-fold, respectively.

#### 3.6.2. Immune-Related Genes Expression in Gills After *C. freundii* Infection

**Figure 7:**
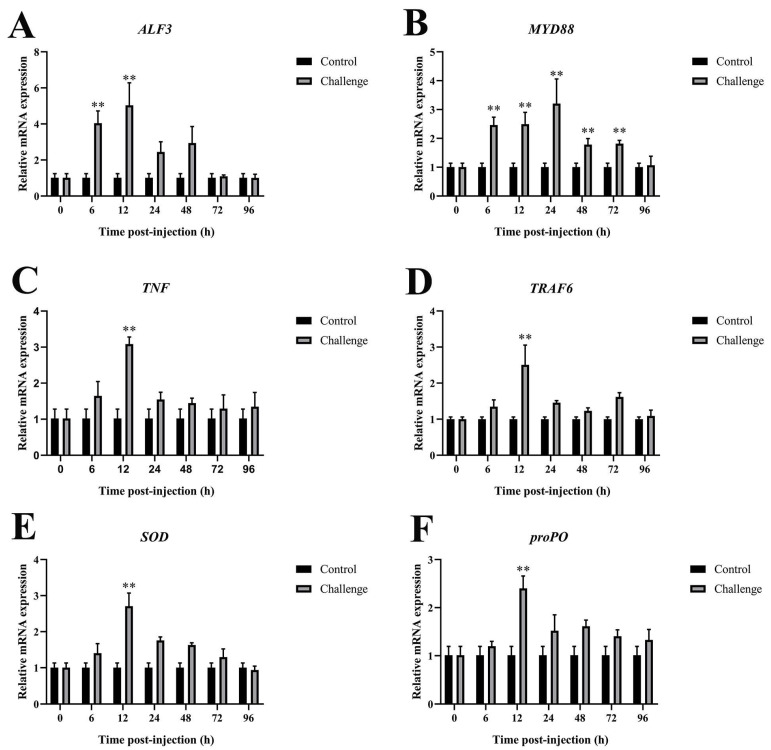
Immune-related gene expression in gills after *C. freundii* infection. (**A**) *ALF3*, (**B**) *MyD88*, (**C**) *TNF*, (**D**) *TRAF6*, (**E**) *SOD*, (**F**) *proPO*. Data presented as mean ± SD, ** *p* < 0.01.

As observed in the gills, qRT-PCR results revealed changes in various immune parameters in the intestines after *C. freundii* infection, with the expression of these genes shown in Figure 7. *ALF3*, *TNF*, *TRAF6*, *SOD*, and *proPO* exhibited their highest expression levels at 12 hpi, with 4.94-fold, 3.03-fold, 2.51-fold, 2.68-fold, and 2.38-fold, respectively. Expression levels of these genes decreased between 12 and 24 hpi (Figure 7A,C–F). *MyD88* was significantly upregulated at 24 hpi, with a maximum level of 3.17-fold. These findings indicate that these immune genes are rapidly upregulated in gill tissues during the initial stages of *C. freundii* infection in *M. rosenbergii* and could serve as potential immune indicators for monitoring disease progression.

#### 3.6.3. Immune-Related Genes Expression in Intestines After *C. freundii* Infection

**Figure 8:**
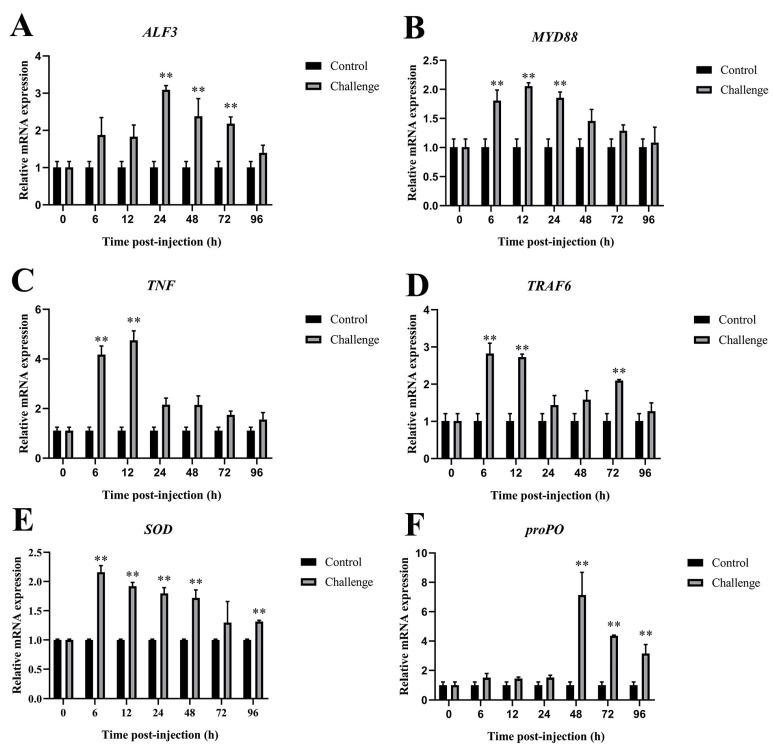
Immune-related gene expression in intestines after *C. freundii* infection. (**A**) *ALF3*, (**B**) *MyD88*, (**C**) *TNF*, (**D**) *TRAF6*, (**E**) *SOD*, (**F**) *proPO*. Data presented as mean ± SD, ** *p* < 0.01.

As shown in Figure 8, significant changes were observed in immune-related gene expression in the intestines of *M. rosenbergii* infected with *C. freundii* compared to those injected with saline solution. *TRAF6* and *SOD* exhibited peak expression levels at 6 hpi, with increases of 2.82-fold and 2.16-fold, respectively, compared to the control. *MyD88* and *TNF* reached their peak expression levels at 12 hpi, showing 2.03-fold and 4.28-fold increases, respectively. *ALF3* peaked at 24 hpi with a 3.06-fold increase, while *proPO* reached its highest expression at 48 hpi with a 7.03-fold increase.

#### 3.6.4. Immune-Related Genes Expression in Hemocytes After *C. freundii* Infection

**Figure 9:**
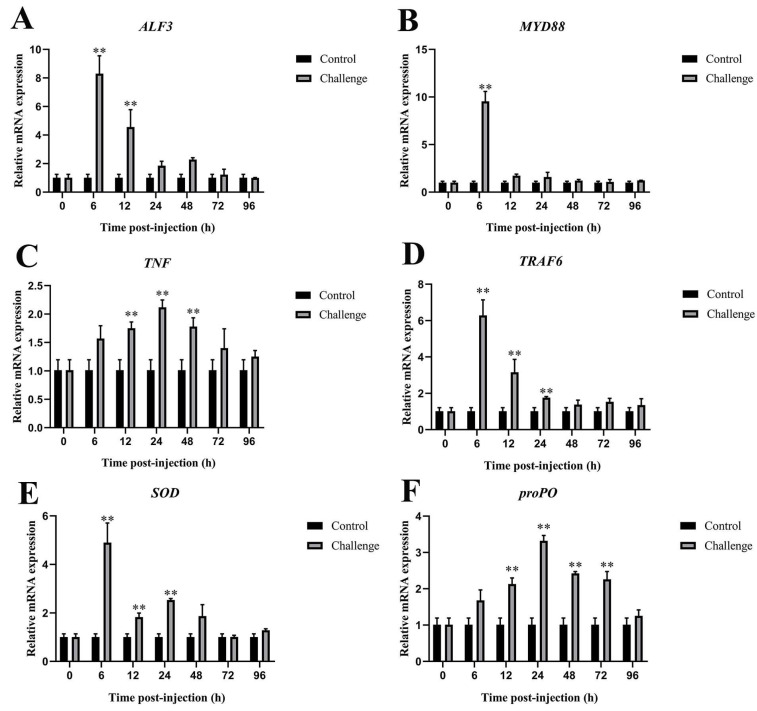
Immune-related gene expression in hemocytes after *C. freundii* infection. (**A**) *ALF3*, (**B**) *MyD88*, (**C**) *TNF*, (**D**) *TRAF6*, (**E**) *SOD*, (**F**) *proPO*. Data presented as mean ± SD, ** *p* < 0.01.

The qRT-PCR results indicated that the expression patterns of 6 immune-related genes in hemocytes differed between the experimental and control groups (Figure 9). *ALF3*, *MyD88*, *TRAF6*, and *SOD* were significantly upregulated at 6 hpi, reached peak levels of 8.14-fold, 9.45-fold, 6.22-fold, and 4.85-fold, respectively, and subsequently decreased from 12 to 48 hpi (Figure 9A,B,D,E). Notably, *TNF* and *proPO* showed significant increases at 24 hpi, with gene expression levels of 2.10-fold and 3.30-fold, respectively (Figure 9C,F).

## 4. Discussion

*Macrobrachium rosenbergii,* an important economic crustacean in China, is experiencing significant production losses worldwide due to disease outbreaks caused by viruses and bacteria, including DIV1 [20], IPV [16], non-O1/O139 *V. cholerae* [8], and *A. veronii* [13], which are exacerbated by its increased density and yield of *M. rosenbergii* farming. In this study, a dominant bacterial strain, FSNM-1, was isolated from mass mortality outbreaks of *M. rosenbergii* that have occurred on farms in Gaoyou County, Yangzhou City, Jiangsu Province. The pathogenicity test indicated that *Citrobacter freundii* led to widespread mortality in *M. rosenbergii,* with the LD_50_ being 1.1 × 10^6^ CFU/mL. Similarly, with an LD_50_ of 1.71 × 10^6^ CFU/mL, *C. freundii* demonstrates a notable level of virulence in crayfish [31]. Additionally, a previous study also showed that the LD_50_ of *C. freundii* for *M. rosenbergii* was 10^4.94^ CFU/g [36], suggesting that *C. freundii* is a highly virulent pathogen for *M. rosenbergii*. Therefore, it can be determined that *C. freundii* was the cause of mortality in *M. rosenbergii* observed on the farm.

Although reports of diseases in aquatic animals, including fish and crustaceans, caused by *C. freundii* are increasing, most studies have concentrated on pathogen isolation and identification. However, few studies have investigated the pathogenic mechanisms of *C. freundii*. The strength of bacterial pathogenicity is primarily determined by virulence factors, which enable pathogenic bacteria to adhere to and evade the host’s defense mechanism [37,38]. To further investigate the virulence of the FSNM-1 strain in *M. rosenbergii*, 11 virulence genes were screened in our study. The results indicated that the FSNM-1 strain carried 7 virulence-related genes, including colonization factor antigens (*cfa1*, *cfa2*), ureases (*ureD*, *ureE*, *ureF*, *ureG*), and outer membrane proteins (*ompX*). Among them, colonization factors are essential for bacterial colonization of host tissues [39]. The colonization factor antigen was a virulent component within the Enterobacteriaceae family, producing an enterotoxin that has been demonstrated to cause death in certain instances [32,40]. *OmpX* is involved in the invasion and adherence of Enterobacteriaceae to host cells, and its homologs have been proposed to contribute to the virulence in various bacterial species [41,42]. Furthermore, Enterobacteriaceae can withstand adverse environments and adhere to hosts by producing urease, which facilitates their proliferation within the host [43].

Invertebrates, including *M. rosenbergii*, rely on their innate immune systems to defend against viral and bacterial invasion. These systems consist of two distinct but coordinated systems: cellular immunity and humoral immunity [44]. The defense of the prawn’s innate immune system against invading pathogens is primarily based on antimicrobial peptides (AMPs), enzymes, and cellular components [45]. AMPs are vital to the invertebrate immune system, serving as a key defense mechanism. It is one of the primary nonspecific immune factors and demonstrates broad-spectrum activity against a range of pathogens, including viruses, bacteria, fungi, and parasites [46]. Anti-lipopolysaccharide factors (ALFs), a subset of AMPs, not only directly eliminate microbes but also modulate host immunity and maintain microbial balance [47]. *ALF8* was reported to play an important role in the immune response of *P. monodon* infected by *V. parahaemolyticus* [48]. In our study, the expression level of *ALF3* increased in the hepatopancreas, gills, intestines, and hemocytes following infection with *C. freundii*, with the highest expression observed in the hemocytes. In addition, the expression of *ALF3* was found to reach its maximum at 6 hpi after *C. freudii* infections in the hemocytes and at 12 hpi in the hepatopancreas, gills, and intestines. *TRAF6* and *MyD88* are essential molecules in the Toll pathway, which is crucial for regulating the innate immune response in invertebrates and providing defense against fungi, Gram-positive bacteria, and viruses [49,50]. It has been reported that *TRAF6* of *Fenneropenaeus penicillatus* could play a positive role against *V. alginolyticus*; the expression of *TRAF6* was increased gradually in the gill, lymphoid organ, and hepatopancreas after *V. alginolyticus* infection and reached the highest level at 12 hpi [51]. In our study, the *TRAF6* expression level was significantly upregulated from 6 to 24 hpi in the hemocytes and reached the maximum at 12 hpi in the gills and intestines and 48 hpi in the hepatopancreas. Moreover, the MyD88-dependent Toll-signaling pathway may be involved in the immune response of *P. monodon* to *V. harveyi* [52]. In our study, the upregulation of *TRAF6* and *MyD88* genes in the hepatopancreas, gills, intestines, and hemocytes proved the activation of the Toll pathway. In addition, *TNF* is a potent inflammatory cytokine crucial for the immune response to pathogenic infections. It plays key roles in apoptosis and phagocytosis of hemocytes, regulates various cellular responses by binding to the tumor necrosis factor receptor (TNFR), and activates immune-related enzymes [53,54]. Reportedly, the *TNF* gene of *Marsupenaeus japonicus* is essential to enhance resistance to *V. nigripulchritudo* [55]. In our study, the *TNF* expression level was significantly upregulated from 12 to 72 hpi in the hepatopancreas and significantly upregulated from 12 to 48 hpi in the hemocytes. The expression of the *TNF* gene was also found to reach the maximum at 12 hpi in the gills and intestines. The upregulated *TNF* gene indicated its significant role in combating *C. freundii* infection. *SOD* is a crucial stress indicator in crustaceans, eliminating excessive *ROS* and playing a vital role in maintaining homeostasis. It hs been reported that the *SOD* expression can rapidly increase following infection with *V. parahaemolyticus* [56]. In this study, the infection of *M. rosenbergii* with *C. freudii* led to a significant upregulation of *SOD* expressions, playing a protective role in resisting *C. freundii* infections. Furthermore, *proPO* is crucial in the immune response of invertebrates, serving not only as a defense mechanism against pathogenic bacterial infection but also as a regulator of normal physiological function [57,58]. It was reported that *proPO* could enhance resistance to WSSV in *P. clarkia,* and it plays an important role in anti-*V. Harvey* in *Penaeus monodon* [18,59]. In our study, the significant upregulation of *proPO* gene expression suggests its potential antimicrobial activity.

## 5. Conclusions

In conclusion, this study identified the *C. freundii* FSNM-1 strain as the bacterial pathogen responsible for the mass mortality of *M. rosenbergii*. The strain’s pathogenic characteristics were elucidated by examining its pathogenicity, virulence factors, and associated genes. Additionally, significant changes were observed in the expression levels of immune-related genes, including *ALF3*, *TNF*, *TRAF6*, *MyD88*, *SOD*, and *proPO* in *M. rosenbergii* with *C. freundii* infection. These findings provide valuable insights into the host’s immune response mechanisms and offer a theoretical foundation for the development of strategies to prevent and control *C. freundii* infections.

## Figures and Tables

**Table 1:** Sequences of primers for virulence genes detection.

Target Gene	Primer Sequence (5′–3′)	Product Size (bp)	Reference
*cfa1*	GCGGTTACTGGAAAGATG	345	CP016762.1
	CGGCGATACTGAAATAGG		
*cfa2*	ATCGTTAGGGTTGGGTGAA	307	CP016762.1
	TTCAGCAATCATTTTTAGCTTCGCC		
*ureD*	GCCAGATGTCACGCATAACG	243	CP016762.1
	GGCTGCCACTGCTGATAGAA		
*ureG*	AGGTTATCGCCACCGCTTTC	119	CP016762.1
	GGTTGCCCGCATACTGCT		
*ureE*	TAACAGGCTTTGGCGAGTAGGA	215	CP016762.1
	CGCCTTGACCACGCTCACT		
*ureF*	ATGCCGCAGAGTTGGCTGTC	300	CP016762.1
	GGAGATTGGCTGGGTGAAAA		
*ompX*	CTACGAATACGGCTCTGC	287	CP016762.1
	ATCGGTTCCTTCACTTACAC		

**Table 2:** Sequences of primers for immune-related genes detection.

Target Gene	Primer Sequence (5′–3′)
*ALF3*	GAACTGCTGTCCAACCCTG
	CCGGATGCTCCTCCGTTATC
*TRAF6*	CGGACGGGAGTTCGATAA
	GGTGCCCACAGGTTGTCT
*MyD88*	GAGTCATGTCAGGCCTACGA
	CACAGCTAGACCCCTCCAAT
*TNF*	TGAGCCGTTTAGCTTGGAGG
	CGCGATTATGAGACCGACCA
*SOD*	TGTCAGAAAGACCACGAA
	GATGCTGGCAACATAAGC
*proPO*	GCAACATTGGCGAACTGA
	GGGAAGGTCTCGACGACT
18S rRNA	CTGTTACGGGTGACGGAGAA
	TCGGAAGAGTCCCGCATT

**Table 3:** Physiological and biochemical characteristics of isolate FSNM-1.

Biochemical Test	FSNM-1	*C. freundii*
Phenylalanine	−	−
Motility	+	+
Sorbose	+	+
O-F test	F	F
Arabinol	−	−
Maltose	+	+
Mannitol	+	+
Mannose	+	+
Sorbitol	+	[+]
Inositol	−	−
Arabinose	+	+
β-galactosidase	+	+
Xylose	+	+
Esculin	+	[+]
Adonitol	−	−
Inulin	+	+
DNase	−	−
Gelatin hydrolysis	−	−
Tartrate utilization	+	+
Nitrate reduction	+	+
Citrate utilization	+	+
Malonate utilization	−	−

Note: “+”, positive; “−”, negative; “F”, fermentative; “[+]”, 76–89% of the strains are positive, data on *C. freundii* from Bergey’s Manual of Systematic Bacteriology.

**Table 4:** The extracellular enzyme and hemolysin activities of the FSNM-1 strain.

Extracellular Products	Clear Zone Diameter/mm
Hemolysin	19.4 ± 1.6
Lecithin	19.6 ± 2.1
Amylase	27.7 ± 2.2
Lipase	14.3 ± 1.2
Gelatinase	17.3 ± 1.5

## Data Availability

Data are contained within the article.
